# Supplementary material for: The Elephant in the Room: A Cross-Sectional Study on the Stressful Psychological Effects of the COVID-19 Pandemic in Mental Healthcare Workers
Source: Brain Sci. 2022 Mar 19;12(3):408. doi: 10.3390/brainsci12030408 (PMC8946286; doi:10.3390/brainsci12030408)
Supplement: Supplementary file 1 [file brainsci-12-00408-s001.zip › brainsci-1589723-supplementary.pdf]

Supplementary Materials

Table S1. Descriptive statistics (absolute and percentages frequencies) on LEC items for overall sample subjects and also stratified for professional roles.

Variables	Overall (N=271)	Nurses (N=86)	Psychiatrists/ Training psychiatrists (N=60)	PRTs/Educators (N=59)	Psychologists/ Psychotherapists (N=44)	Other mental health professionals (N=22)
**LEC1 natural disaster**						
Not sure, n	4	2	0	0	1	1
0, n (%)	172 (64.4%)	49 (58.3%)	44 (73.3%)	43 (72.9%)	28 (65.1%)	8 (38.1%)
1, n (%)	95 (35.6%)	35 (41.7%)	16 (26.7%)	16 (27.1%)	15 (34.9%)	13 (61.9%)
**LEC2 fires explosions**						
Not sure, n	8	3	2	1	1	1
0, n (%)	220 (83.7%)	68 (81.9%)	53 (91.4%)	44 (75.9%)	40 (93.0%)	15 (71.4%)
1, n (%)	43 (16.3%)	15 (18.1%)	5 (8.6%)	14 (24.1%)	3 (7.0%)	6 (28.6%)
**LEC3 transportation accident**						
Not sure, n	6	1	2	2	1	0
0, n (%)	147 (55.5%)	44 (51.8%)	33 (56.9%)	34 (59.6%)	26 (60.5%)	10 (45.5%)
1, n (%)	118 (44.5%)	41 (48.2%)	25 (43.1%)	23 (40.4%)	17 (39.5%)	12 (54.5%)
**LEC4 serious accident**						
Not sure, n	10	2	2	3	3	0
0, n (%)	217 (83.1%)	68 (81.0%)	49 (84.5%)	48 (85.7%)	37 (90.2%)	15 (68.2%)
1, n (%)	44 (16.9%)	16 (19.0%)	9 (15.5%)	8 (14.3%)	4 (9.8%)	7 (31.8%)
**LEC5 exposure toxic substances**						
Not sure, n	10	3	2	3	1	1
0, n (%)	250 (95.8%)	79 (95.2%)	56 (96.6%)	54 (96.4%)	41 (95.3%)	20 (95.2%)
1, n (%)	11 (4.2%)	4 (4.8%)	2 (3.4%)	2 (3.6%)	2 (4.7%)	1 (4.8%)

Variables	Overall (N=271)	Nurses (N=86)	Psychiatrists/ Training psychiatrists (N=60)	PRTs/Educators (N=59)	Psychologists/ Psychotherapists (N=44)	Other mental health professionals (N=22)
**LEC6 physical assault**						
Not sure, n	3	0	1	1	0	1
0, n (%)	165 (61.6%)	46 (53.5%)	35 (59.3%)	37 (63.8%)	33 (75.0%)	14 (66.7%)
1, n (%)	103 (38.4%)	40 (46.5%)	24 (40.7%)	21 (36.2%)	11 (25.0%)	7 (33.3%)
**LEC 7 assault with weapon**						
Not sure, n	7	1	1	3	1	1
0, n (%)	233 (88.3%)	72 (84.7%)	52 (88.1%)	51 (91.1%)	39 (90.7%)	19 (90.5%)
1, n (%)	31 (11.7%)	13 (15.3%)	7 (11.9%)	5 (8.9%)	4 (9.3%)	2 (9.5%)
**LEC8 sexual assault**						
Not sure, n	9	4	0	5	0	0
0, n (%)	249 (95.0%)	79 (96.3%)	56 (93.3%)	50 (92.6%)	43 (97.7%)	21 (95.5%)
1, n (%)	13 (5.0%)	3 (3.7%)	4 (6.7%)	4 (7.4%)	1 (2.3%)	1 (4.5%)
**LEC9 other unwanted sexual experience**						
Not sure, n	12	4	4	3	0	1
0, n (%)	233 (90.0%)	76 (92.7%)	47 (83.9%)	51 (91.1%)	38 (86.4%)	21 (100.0%)
1, n (%)	26 (10.0%)	6 (7.3%)	9 (16.1%)	5 (8.9%)	6 (13.6%)	0 (0.0%)
**LEC10 exposure warzone**						
Not sure, n	7	3	1	1	1	1
0, n (%)	255 (96.6%)	78 (94.0%)	57 (96.6%)	57 (98.3%)	43 (100.0%)	20 (95.2%)
1, n (%)	9 (3.4%)	5 (6.0%)	2 (3.4%)	1 (1.7%)	0 (0.0%)	1 (4.8%)
**LEC11 captivity**						
Not sure, n	4	2	0	1	0	1
0, n (%)	265 (99.3%)	84 (100.0%)	59 (98.3%)	57 (98.3%)	44 (100.0%)	21 (100.0%)
1, n (%)	2 (0.7%)	0 (0.0%)	1 (1.7%)	1 (1.7%)	0 (0.0%)	0 (0.0%)

Variables	Overall (N=271)	Nurses (N=86)	Psychiatrists/ Training psychiatrists (N=60)	PRTs/Educators (N=59)	Psychologists/ Psychotherapists (N=44)	Other mental health professionals (N=22)
**LEC 12 illness injury**						
Not sure, n	4	3	0	1	0	0
0, n (%)	203 (76.0%)	64 (77.1%)	47 (78.3%)	48 (82.8%)	28 (63.6%)	16 (72.7%)
1, n (%)	64 (24.0%)	19 (22.9%)	13 (21.7%)	10 (17.2%)	16 (36.4%)	6 (27.3%)
**LEC13 severe human suffering**						
Not sure, n	0	0	0	0	0	0
0, n (%)	142 (52.4%)	42 (48.8%)	28 (46.7%)	36 (61.0%)	22 (50.0%)	14 (63.6%)
1, n (%)	129 (47.6%)	44 (51.2%)	32 (53.3%)	23 (39.0%)	22 (50.0%)	8 (36.4%)
**LEC14 sudden violent death**						
Not sure, n	2	0	0	2	0	0
0, n (%)	232 (86.2%)	68 (79.1%)	52 (86.7%)	50 (87.7%)	43 (97.7%)	19 (86.4%)
1, n (%)	37 (13.8%)	18 (20.9%)	8 (13.3%)	7 (12.3%)	1 (2.3%)	3 (13.6%)
**LEC15 sudden accidental death**						
Not sure, n	3	0	1	2	0	0
0, n (%)	249 (92.9%)	79 (91.9%)	57 (96.6%)	53 (93.0%)	40 (90.9%)	20 (90.9%)
1, n (%)	19 (7.1%)	7 (8.1%)	2 (3.4%)	4 (7.0%)	4 (9.1%)	2 (9.1%)
**LEC16 serious injury death you caused**						
Not sure, n	5	2	1	2	0	0
0, n (%)	261 (98.1%)	84 (100.0%)	58 (98.3%)	54 (94.7%)	43 (97.7%)	22 (100.0%)
1, n (%)	5 (1.9%)	0 (0.0%)	1 (1.7%)	3 (5.3%)	1 (2.3%)	0 (0.0%)
**LEC17 any stressful event**						
Not sure, n	8	3	1	2	2	0
0, n (%)	186 (70.7%)	56 (67.5%)	40 (67.8%)	43 (75.4%)	28 (66.7%)	19 (86.4%)
1, n (%)	77 (29.3%)	27 (32.5%)	19 (32.2%)	14 (24.6%)	14 (33.3%)	3 (13.6%)

“Not sure” include the following answer: (e) you’re not sure if it fits. In further analysis, we treated this response as missing value since it is not informative, dichotomizing the LEC items; 0: Include the following answers: (c) you learned about it happening to a close family member or close friend; (d) you were exposed to it as part of your job (for example, paramedic, police, military, or other first responder); (f) it doesn’t apply to you; 1: Include the following answers: (a) it happened to you personally; (b) you witnessed it happen to someone else.

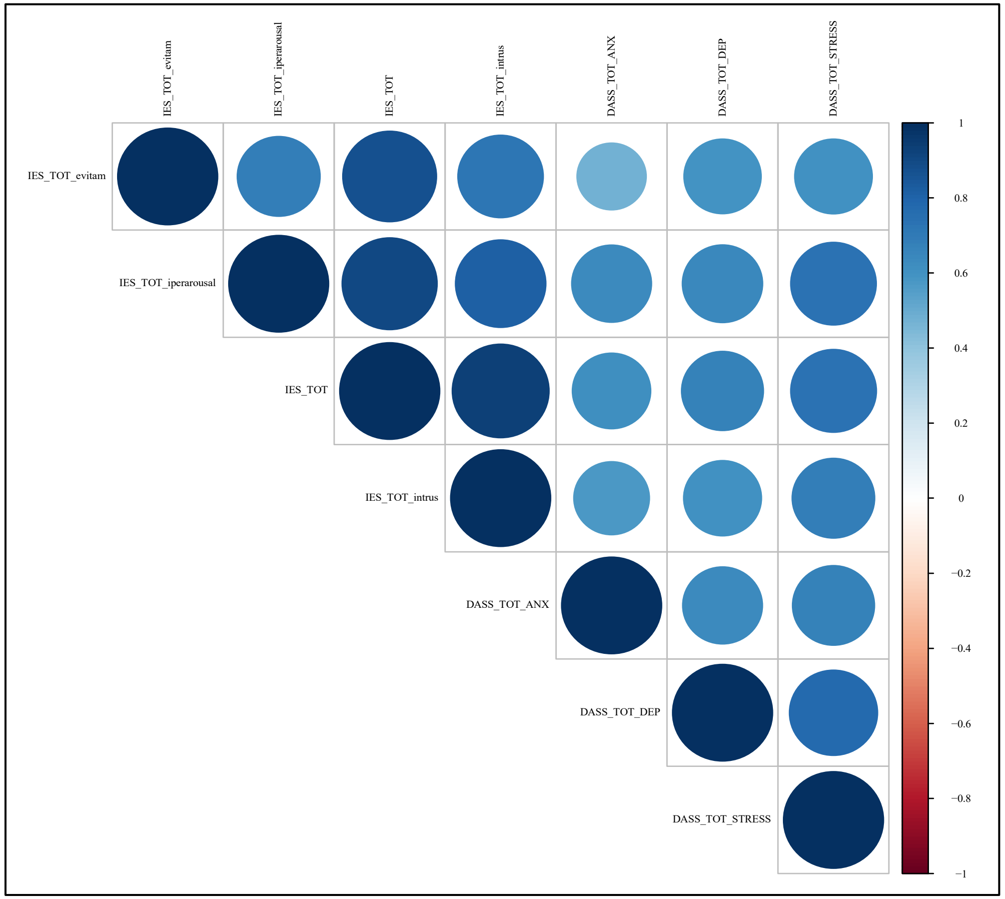

Figure S1. Correlation plot between IES and DASS dimensions. Blue circles correspond to positive correlation between couple of variables. Diameters and colors intensity are proportional to the magnitude of Spearman coefficients and. Since there are not black crosses on them, correlations are all significantly different from zero (p-values<0.05).

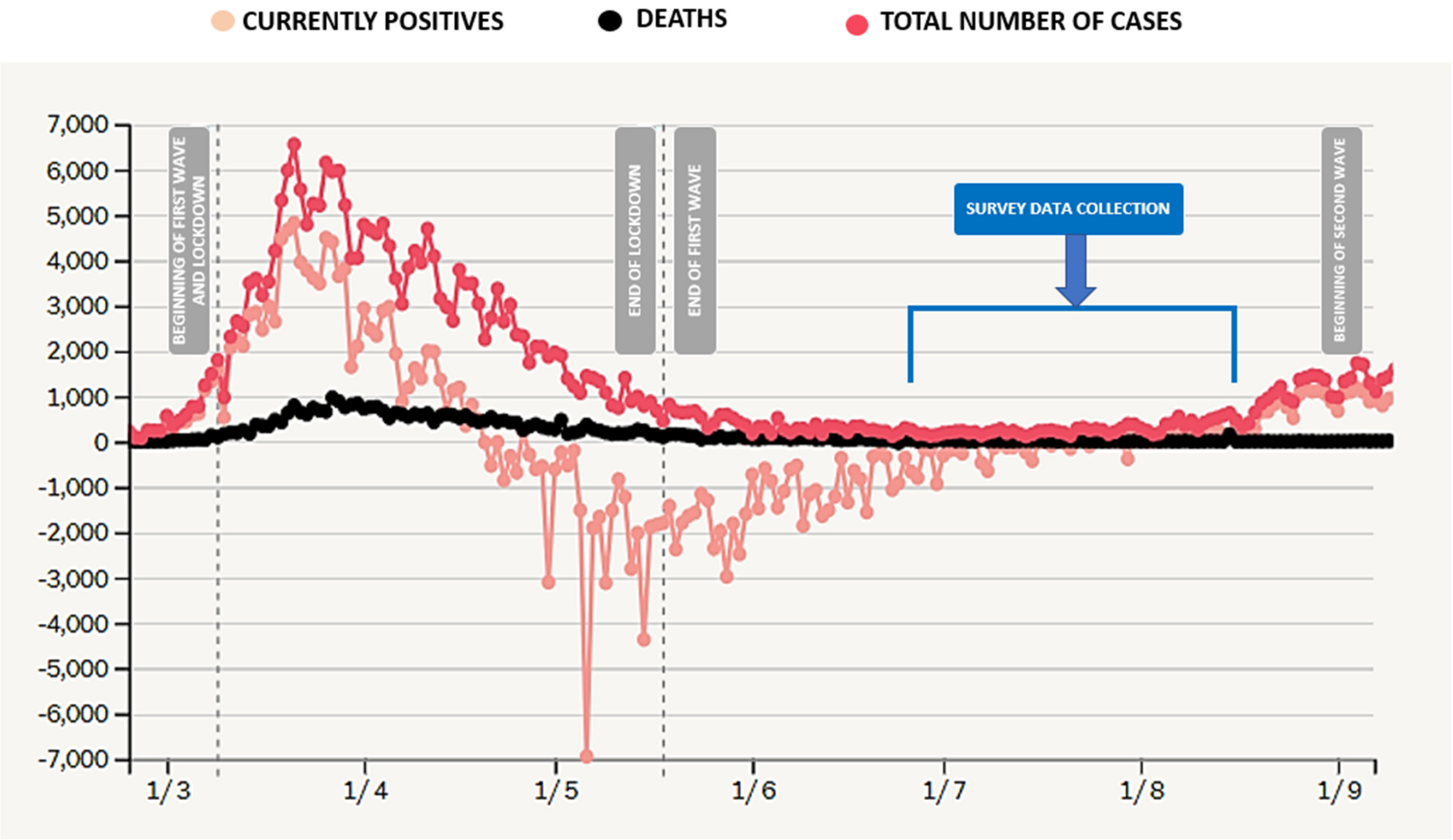

Figure S2. Graphics representing the first and second waves of COVID-19 infection in Italy in 2020.

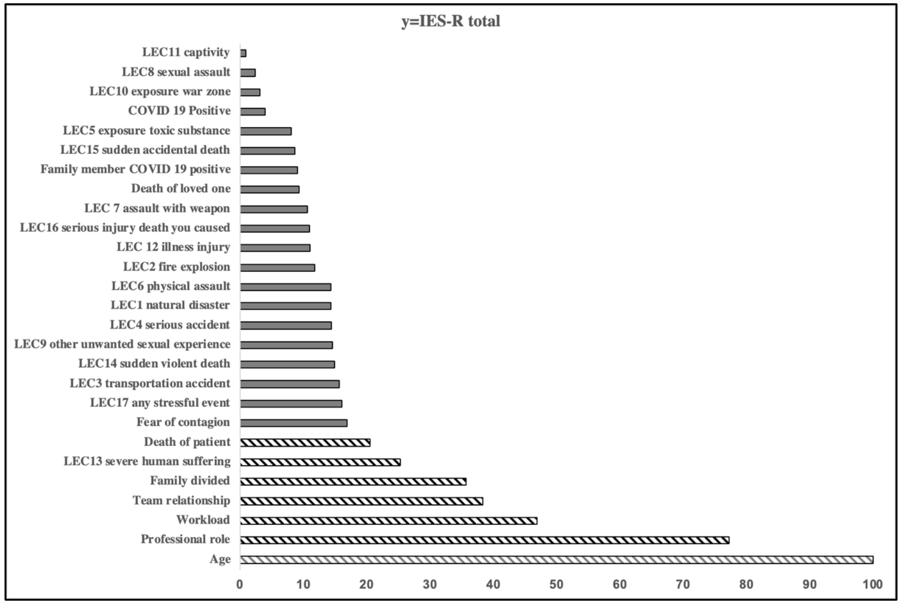
y=IES-R total
LEC11 captivity
LEC8 sexual assault
LEC10 exposure war zone
COVID 19 Positive
LEC5 exposure toxic substance
LEC15 sudden accidental death
Family member COVID 19 positive
Death of loved one
LEC 7 assault with weapon
LEC16 serious injury death you caused
LEC 12 illness injury
LEC2 fire explosion
LEC6 physical assault
LEC1 natural disaster
LEC4 serious accident
LEC9 other unwanted sexual experience
LEC14 sudden violent death
LEC3 transportation accident
LEC17 any stressful event
Fear of contagion
Death of patient
LEC13 severe human suffering
Family divided
Team relationship
Workload
Professional role
Age
0 10 20 30 40 50 60 70 80 90 100

S3.1

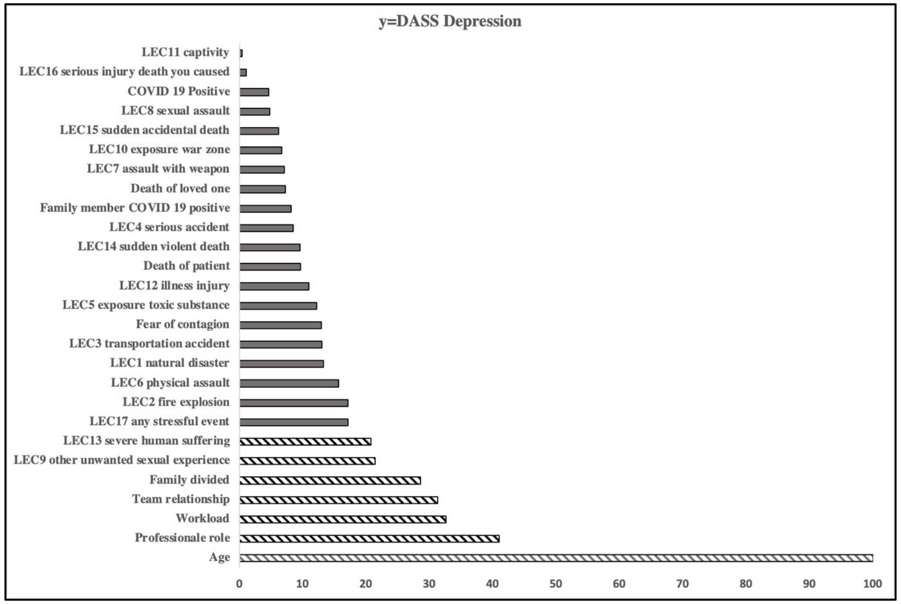
y=DASS Depression
LEC11 captivity
LEC16 serious injury death you caused
COVID 19 Positive
LEC8 sexual assault
LEC15 sudden accidental death
LEC10 exposure war zone
LEC7 assault with weapon
Death of loved one
Family member COVID 19 positive
LEC4 serious accident
LEC14 sudden violent death
Death of patient
LEC12 illness injury
LEC5 exposure toxic substance
Fear of contagion
LEC3 transportation accident
LEC1 natural disaster
LEC6 physical assault
LEC2 fire explosion
LEC17 any stressful event
LEC13 severe human suffering
LEC9 other unwanted sexual experience
Family divided
Team relationship
Workload
Professionale role
Age
0 10 20 30 40 50 60 70 80 90 100

S3.2

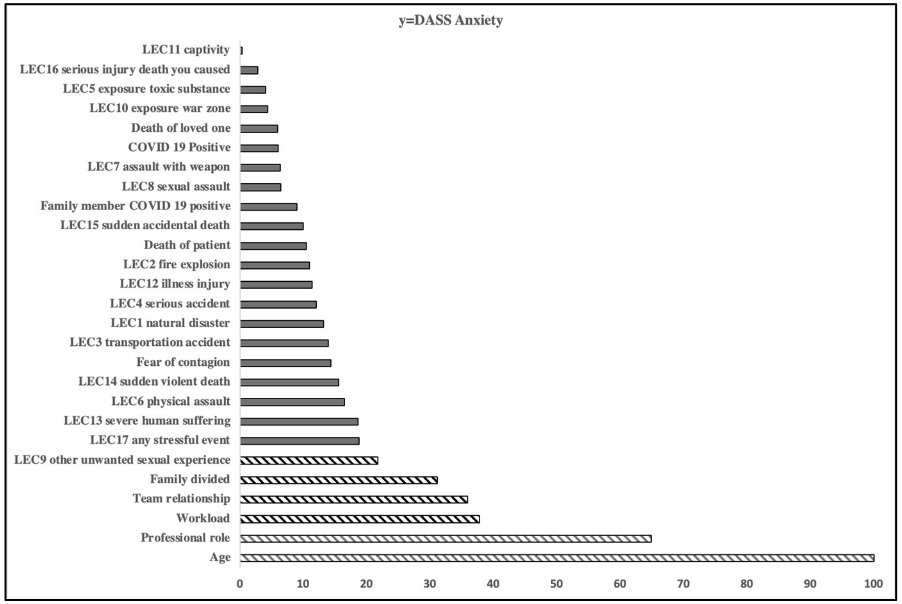

S3.3

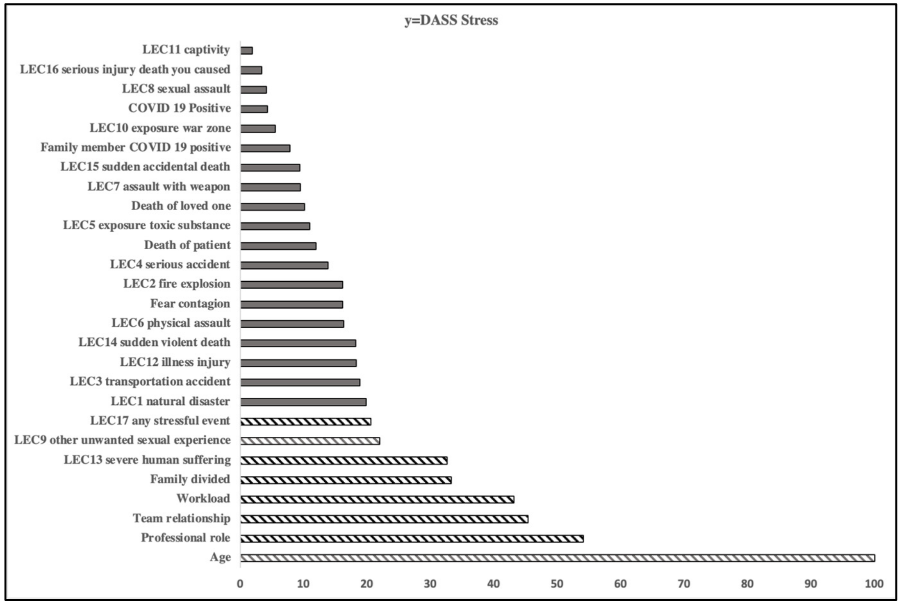

S3.4

Figure S3. Relative Variable Importance (relVIM) extracted from a Random Forest (RF) identifies variables that have a strong impact on the predictions of the IES-R total (1), DASS Depression (2), DASS Anxiety (3) and DASS Stress (4) scores. The black and white striped bars highlight variables whose relVIM>20 whereas the remaining variables (grey bars) have a marginal role in the model.

Invitation text

Invitation text put into circulation, directed to mental healthcare professionals, through emails and WhatsApp links:

Carissima/o
Mi permetto di inviarti il link (https://forms.gle/LsMoJsjewvVXRn1E6) di questa survey sviluppata da me con alcuni colleghi al fine di poter valutare lo stress post emergenza Covid negli operatori della salute mentale. Ti chiederei pertanto la disponibilità ad impiegare 15 minuti del tuo tempo per compilarla, sarebbe per tutti noi del settore della salute mentale molto importante. I dati raccolti serviranno ad indagare gli effetti degli elevati livelli di stress, causati dalla pandemia Covid-19, su tutto il personale dei servizi di salute mentale, mettendo in risalto le difficoltà e le problematiche incontrate. Lo studio vorrebbe inoltre supportare il ruolo degli operatori sanitari della salute mentale, producendo risultati che possano indirizzare azioni e politiche di sostegno agli stessi professionisti. Ti chiedo inoltre, sempre che non sia un problema, la cortesia di inoltrarla a tutti gli operatori che conosci e che a vario titolo (psichiatri, psicologi, infermieri, oss, TeRP, assistenti sociali, terapisti occupazionali, ecc.) operano nell'ambito della salute mentale. Grazie di cuore. Buona giornata.

Dearest,
I would like to send you the following link (https://forms.gle/LsMoJsjewvVXRn1E6) of this survey developed by me along with some colleagues in order to evaluate post-Covid emergency stress in mental health workers. I would therefore ask you if you could spend 15 minutes of your time to fill it out, since it would be very important for all of us in the mental healthcare sector. The collected data will be used to investigate the effects of high stress level caused by the Covid-19 pandemic on all mental health service staff, highlighting the difficulties and problems encountered. The study would be useful to support the role of mental health professionals and its results could direct actions and policies to support these workers. I also ask you, provided that it is not a problem, the courtesy of forwarding it to all the professionals you know who work in the field of mental health, including psychiatrists, psychologists, nurses, social workers, occupational therapists, etc. Thank you very much. Have a nice day.

Survey Introduction:

SURVEY OPERATORI DELLA SALUTE MENTALE
Cara/o Collega,

Ti invitiamo a partecipare al nostro studio finalizzato a rilevare lo stress correlato all’emergenza COVID-19 negli operatori della salute mentale, in quanto tale emergenza può aver complicato la gestione quotidiana inerente a ciascun ruolo e ambito lavorativo in tale settore che già solitamente si trova a fronteggiare situazioni complesse con un’utenza al tempo stesso fragile e talvolta di difficile gestione.

Tale indagine includerà sia domande generiche, sia domande relative al tuo stato psicologico in questo momento di emergenza e richiederà circa 15 minuti per la sua compilazione. Sarà possibile rispondere a tale survey per un tempo di un mese dalla ricezione del link per motivi metodologici di uniformità e validità dei dati raccolti.

Ti chiediamo un piccolo sforzo nella compilazione in quanto ciascun contributo sarà fondamentale nel capire questo fenomeno e nel poter delineare risposte adeguate nel supporto degli operatori della salute mentale.

Lo studio è stato approvato dal Comitato Etico ASST Spedali Civili di Brescia (NP4172).

La tua partecipazione è completamente volontaria. Non ci sono rischi prevedibili associati a questo studio. Tuttavia, se ti sentissi a disagio nel rispondere ad una qualsiasi domanda, puoi interrompere l'indagine in qualunque momento.

Il questionario è anonimo e rispetta tutti gli standard etici della ricerca. In particolare, la protezione dei dati personali è tutelata sia dalla specifica normativa (D.L. 101/18) sia dalle persone responsabili del progetto di ricerca.

Ti ringraziamo anticipatamente per il tuo tempo e per la tua collaborazione.

Ho letto, compreso e acconsento alla partecipazione allo studio?
Sì
No
Consenso informato: puoi iniziare l'indagine selezionando la casella “Acconsento” e cliccando successivamente sul pulsante “Avanti” in basso.
Acconsento
Non acconsento

MENTAL HEALTH WORKERS SURVEY
Dear Colleague,

We would like to invite you to participate in our study aimed at detecting stress related to the COVID-19 emergency in mental healthcare workers, since this emergency may have complicated the daily management inherent in each role and work environment in this sector, that already usually faces complex situations with fragile and sometimes difficult to manage users.
This survey includes both general questions and others related to your psychological state in this time of emergency and will take about 15 minutes to be filled. It will be possible to answer to this survey for a period of one month from the receipt of this link for methodological reasons that requires uniformity and validity of the collected data.
We would ask you for a careful compilation of the survey since each contribution will be fundamental in understanding this phenomenon and in outlining adequate responses to support mental healthcare professionals.
This study was approved by the ETHICS Committee of the ASST Spedali Civili of Brescia (NP4172).
Your participation is completely voluntary. There are no foreseen risks associated with the participation in this study. However, if you feel uncomfortable answering any question, you can quit at any time.
This questionnaire is anonymous and meets all ethical standards in research. In particular, the protection of personal data is assured both by the specific legislation (Legislative Decree 101/18) and by the coordinators of this research project.
We thank you in advance for your time and for your cooperation.

Have I read, understood and consent to participation in the study? Yes / No
Informed consent: You can start the survey by checking the "I agree" box and then clicking on the "Next" button below. I agree / I do not agree.
